# Pharmacokinetic Comparisons of Naringenin and Naringenin-Nicotinamide Cocrystal in Rats by LC-MS/MS

**DOI:** 10.1155/2020/8364218

**Published:** 2020-04-01

**Authors:** Dan Xu, Gui-Qiu Zhang, Ting-Ting Zhang, Bo Jin, Chen Ma

**Affiliations:** Institute of Materia Medica, Chinese Academy of Medical Sciences and Peking Union Medical College, Beijing 100050, China

## Abstract

Naringenin (NAR), 4′,5,7-trihydroxydihydroflavone, has a wide range of pharmacological activities but shows poor water solubility and low bioavailability. The pharmacokinetics and bioavailability of naringenin-nicotinamide cocrystal (NAR-NCT), which offers improved solubility, were evaluated in this study. Rats were orally administered NAR, a physical mixture of naringenin and nicotinamide (NAR + NCT), and NAR-NCT. The relative bioavailability of NAR-NCT was 175.09% of NAR, C_max_ was 8.43 and 2.06 times of NAR and NAR + NCT, respectively, Tmax was advanced from 0.49 h to 0.09 h, CL was decreased from 91.1 L/h/kg to 49.1 L/h/kg, and *t*_1/2_ was increased from 5.37 h to 8.24 h, highlighting its rapid absorption and slow elimination. This study showed that NAR-NCT could improve the bioavailability of NAR.

## 1. Introduction

**Figure 1:**
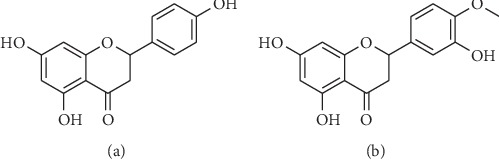
Chemical structure: (a) naringenin and (b) hesperetin.

Naringenin (NAR), 4′,5,7-trihydroxydihydroflavone (Figure 1), is a dihydroflavonoid widely found in grapefruit, grapes, and citrus fruits of the Rutaceae family. It is of great interest because of its rich pharmacological activity including antioxidation [1, 2], antitumour [3, 4], anti-inflammatory [5, 6], antiradiation [7, 8], and immunoregulatory effects [9] as well as having effects on cough and phlegm [10], which has resulted in its wide use in the medicine [11], food, and cosmetics industries. Although it has a variety of pharmacological activities, studies have shown that NAR is poorly soluble in water [12] and has low bioavailability in vivo [13, 14].

Cocrystal is defined as the crystal of combination with active pharmaceutical ingredient and cocrystal former. It with specific physical and chemical properties can form through weak noncovalent interactions, for example, hydrogen bonding [15]. Compared with the parent compound, active pharmaceutical ingredients in the form of cocrystal can improve different properties [16], such as altered stability [17, 18], solubility [19], and bioavailability [20, 21]. Previously, we prepared naringenin-nicotinamide cocrystal (NAR-NCT) to improve the solubility of NAR in different pH buffer solutions [22]. Pharmacokinetic comparisons of NAR and NAR-NCT after oral administration in rats were investigated in this work. After oral administration of NAR, the physical mixture (NAR + NCT), and NAR-NCT, and the plasma concentrations of NAR were determined by LC-MS/MS. NAR-NCT can improve the bioavailability of NAR and result in faster absorption and slower elimination.

## 2. Experimental

### 2.1. Materials

Naringenin (>96%) and nicotinamide (>98%, NCT) were provided by Bailingwei Technology (Beijing, China). Internal standard (IS), hesperetin (>98%, Figure 1), was provided from YIFEI (Shanghai, China). Acetic acid was provided from TEDIA (Fairfield, OH, USA). Methanol (HPLC grade), ethyl acetate (GC grade), and other chemicals were provided from ANPEL (Shanghai, China).

### 2.2. Preparation and Characterization of NAR-NCT

The cocrystal was prepared by solvent evaporation. 50 mg of NAR and 45 mg of NCT (at a molar ratio of NAR and NCT of 1 : 2) were mixed with ethyl acetate (10 mL). After ultrasonic dissolution, the mixtures were heated at 40°C for 4 hours [22]. Differential scanning calorimetry (DSC) was performed on a DSC-TGA-STARe thermal analyzer (Mettler, Toledo, Switzerland). X-Ray powder diffraction (XRPD) was used to analyse the changes of NAR-NCT. XRPD was conducted with a D8 ADVANCE powder diffractometer (Bruker, Karlsruhe, Germany). Infrared spectroscopy (IR) was performed on a Spectrometer 400 Fourier-infrared spectrometer (PerkinElmer, Waltham, America). The NAR-NCT was kept at 60°C, at a relative humidity of 90 ± 5%, under light of 5000 Lx for 10 days and after 6 months at room temperature.

### 2.3. Instrumentation

The chromatographic separation was carried out by a Shimadzu UFLC-20AD XR system (Tokyo, Japan). The column was Shim-Pack C18-ODS column (75 mm × 3 mm, 2.3 *μ*m) at 40°C and 0.3 mL/min. The mobile phase constituted 0.2% acetic acid-water (v/v, A) and methanol (B). The injection volume was 3 *μ*L. The chromatographic program was as follows: gradient elution, 0–3 min, 60% B; 3–3.5 min, 60–90% B; 3.5–6.5 min, 90% B; 6.5–7 min, 90–60% B; and 7–10 min, 60% B.

AB SCIEX Qtrap 5500 (MDS-Sciex, Concord, Canada) with a negative mode electrospray ionization (ESI) interface was equipped with Shimadzu UFLC-20AD XR. Multiple reaction monitoring was used to analyse samples. Mass spectrometry parameters are shown in Table 1.

**Figure 2:**
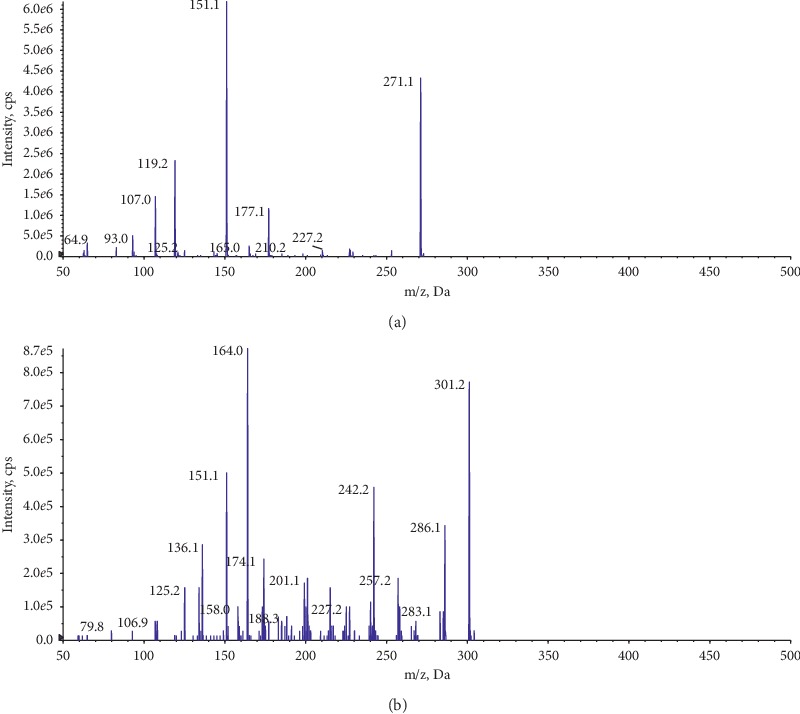
Mass spectrometry of the product ion: (a) NAR and (b) IS.

Secondary mass spectrometry enhanced product ion (EPI) scan was used to obtain a high-quality MS/MS spectrum on a specific ion. This scan type was performed on NAR and IS. The product ion mass spectra about NAR and IS are recorded in Figure 2. The ratio of peak area about NAR to IS was used to determine concentrations of NAR. Data acquisition and analysis were calculated by Analyst 1.6 software. After that, to minimize the contamination of the sample in the mass spectrometer, after the peaks of NAR and IS had eluted, the remaining components were sent from the LC to the waste valve instead of proceeding through the MS system.

### 2.4. Standard Working Solutions and Internal Standard Solution

NAR was dissolved in methanol to obtain a 102.6 *μ*g/mL stock solution. It was diluted with methanol to obtain standard working solutions of 10.3, 30.8, 102.6, 513, 1026, 2052, 4104, 6156, and 8208 ng/mL. Hesperidin was dissolved in methanol (300 ng/mL) as an internal standard solution.

### 2.5. Calibration Standards and Quality Control (QC) Samples

The calibration standards of NAR at a range of 1.03 to 821 ng/mL, which were prepared from blank plasma and NAR standard working solutions.

QC samples were formulated at low, medium, and high concentrations (LQC, MQC, and HQC) corresponding to NAR of 3.08, 410, and 616 ng/mL, respectively, following the procedure described above. The samples were protected from light.

### 2.6. Pretreatment of the Plasma Samples

Plasma (100 *μ*L) was combined with 10 *μ*L of 300 ng/mL internal standard solution in a centrifuge tube, and the mixture was vortexed for 5 min. Then, samples were mixed with ethyl acetate (300 *μ*L), and mixtures were vortex-mixed for 5 min. After that, mixtures were centrifuged at 8000 r/min for 2 min. All supernatants were pipetted into a new tube and dried at 40°C under flowing nitrogen. And then, residues were reconstituted with methanol (100 *μ*L) and vortex-mixed for 1 min.

### 2.7. Method Validations

All method validations complied with US Food and Drug Administration guidelines [23].

#### 2.7.1. Specificity

To demonstrate that substances being measured were intended analytes and endogenous substances and other metabolites do not interfere with the assay, method specificity was established firstly. This analysis involved blank plasma samples (different sources), the mixture of NAR standard solution and blank plasma, and plasma samples after oral administration (*n* = 6).

#### 2.7.2. The Calibration Curve and Lower Limit of Quantification (LLOQ)

Six blank rat plasma samples were combined with the NAR standard working solutions to give final NAR plasma concentrations of 1.03, 10.3, 51.3, 103, 205, and 821 ng/mL. As described in Section 2.6, the NAR plasma concentration (ng/mL) was taken as the abscissa, and the ratio of the NAR to IS peak areas was taken as the ordinate and by a weighted least-squares linear regression (the weighting factor 1/*X*^2^) to obtain the calibration curve.

The lowest concentration of analyte that can be reliably quantified in the sample was the LLOQ. Six standard plasma samples containing NAR were analysed as described in Section 2.6.

At the LLOQ, the relative standard deviation (RSD, %) was ≤20%, the relative error (RE, %) was required to be within ±20%.

#### 2.7.3. Precision and Accuracy

NAR plasma standard samples were prepared at the LLOQ, LQC, MQC, and HQC concentrations (1.03, 3.08, 410, and 616 ng/mL), according to Section 2.6. Six samples were prepared at each concentration. The concentration of each sample was determined on the same day and three consecutive days to assess the intra- and interday precision and accuracy.

The acceptance criteria were RSD (%) was ≤15%, RE (%) was limited to ±15% for the intra- and interday, except at the LLOQ, where RSD (%) should be ≤20% and RE (%) within ±20%.

#### 2.7.4. Extraction Recovery (*R*)

NAR plasma standard samples were prepared at the LLOQ, LQC, MQC, and HQC concentrations (1.03, 3.08, 410, and 616 ng/mL). Then, internal standard solution (10 *μ*L) was added to the mixture, vortexed for 5 min, and added ethyl acetate (300 *μ*L). After that, the samples were centrifuged at 8000 r/min for 2 min. All supernatant was transferred to a new tube. All of them were concentrated to dryness under flowing nitrogen, and the residue was redissolved in methanol (200 *μ*L) to prepare it for analysis. The peak area of NAR (As) was recorded.

Blank plasma samples (100 *μ*L) from different sources were mixed with ethyl acetate (300 *μ*L). The samples were vortexed for 5 min, then centrifuged for 2 min at 8000 r/min, and then all supernatant was removed for analysis. Aliquots 10 *μ*L of the standard solutions (10.3, 30.8, 4104, and 6156 ng/mL) and 10 *μ*L of 300 ng/mL internal standard solution were added. All of them were concentrated to dryness under flowing nitrogen, and the residues were redissolved in methanol (200 *μ*L) for analysis. The peak area of NAR (As′) was recorded.

The extraction recovery (%) of NAR was obtained by substituting As and As′ into the following equation:

(1) $$\begin{array}{c} R\left(\%\right)=\frac{\text{As}}{{\text{As}}^{\prime }}\times 100\%. \end{array}$$

The recovery of the IS was investigated similarly.

#### 2.7.5. Matrix Effect (ME)

The standard solutions (10 *μ*L, at concentrations of 10.3, 30.8, 4104, and 6156 ng/mL) were combined with the internal standard solution (10 *μ*L, 300 ng/mL) in a centrifuge tube. The mixture was vortexed (5 min), dried at 40°C under flowing nitrogen, and redissolved in methanol (200 *μ*L). The supernatant was then analysed. The peak area of NAR (Bs) was recorded.

Blank plasma samples (100 *μ*L), from different sources, were each mixed with ethyl acetate (300 *μ*L), and the mixture was vortexed (5 min) and centrifuged (8000 r/min, 2 min). All supernatant of each sample was removed to a new tube, and standard solution (10 *μ*L, at concentrations of 10.3, 30.8, 4104, and 6156 ng/mL) was added to the internal standard solution (10 *μ*L, 300 ng/mL). The sample was dried at 40°C under flowing nitrogen. Methanol (200 *μ*L) was used to reconstitute in the residue. The peak area of NAR (Bs′) was recorded.

The matrix effect (%) on NAR was calculated according to the following formula:

(2) $$\begin{array}{c} \text{ME}\left(\%\right)=\frac{{\text{Bs}}^{\prime }}{\text{Bs}}\times 100\%. \end{array}$$

The ME of the IS was investigated.

#### 2.7.6. Stability

Analyte's stability in the matrix at the LLOQ, LQC, MQC, and HQC under different conditions was investigated. The evaluated conditions were 24 h short-term stability at room temperature (25 ± 3°C), freeze-thaw cycles three times, long-term stability under −80°C storage 21 days, and room temperature in an automatic sampler for 24 h.

The acceptance criteria for the RSD (%) was ≤15%, RE (%) was limited to ±15%, except at the LLOQ, where RSD(%) was ≤20% and RE(%) within ±20%.

#### 2.7.7. Dilution Reliability

The dilution reliability of NAR in plasma must be examined because of the high concentrations (beyond the calibration curve) of some drugs in the plasma. The plasma sample (NAR, 6156 ng/mL) was diluted with blank plasma 10 times and analysed according to Section 2.6. After sample analysis, the ratio of peak area of NAR to IS was compared to the calibration curve, and RE and RSD values were also calculated (*n* = 6). Accuracy of the concentration determined following dilution was required to be between 85% and 115%, and the precision should be within 15%.

### 2.8. Pharmacokinetic Study

Eighteen SD rats (200 ± 20 g, 8 weeks, half male and half female) were obtained from National Institutes for Food and Drug Control (Beijing, China). The room environment was about 22 ± 2°C, the humidity of 50 ± 10%, light and dark recycle for 12 h. Before the experiment, the rats can be given water freely but were fasted for 12 h. All experiments were following the National Institutes of Health Guide for the Care and Use of Laboratory Animals [24].

There were three groups for oral administration. The first group was administered NAR (30 mg/kg). The next group was administered NAR + NCT, in which the molar ratio of NAR and NCT was 1 : 2, in terms of NAR 30 mg/kg. The last group was administered NAR-NCT (57 mg/kg), which was equal to the NAR dose of 30 mg/kg. These were ground with 0.5% sodium carboxymethyl cellulose, at a dose of 1 mL/100 g. At 0.05, 0.083, 0.167, 0.333, 0.5, 1, 2, 4, 8, 10, 12, and 24 h after administration, 0.5 mL serial blood samples were obtained from the ocular vein into centrifuge tubes. The samples were centrifuged at 10000r/min (10 min, 4°C), and after that, all supernatant of each sample was removed to store at −80°C.

### 2.9. Data Analysis

Noncompartmental modelling was evaluated for pharmacokinetic parameters through DAS Software (China State Drug Administration, version 3.2.4).

The following equation was used to calculate the relative oral bioavailability (Fr, %) in rats:

(3) $$\begin{array}{c} \text{Fr}\%=\frac{{\text{AUC}}_{\text{NAR}‐\text{NCT}}\times {\text{dose}}_{\text{NAR}}}{{\text{AUC}}_{\text{NAR}}\times {\text{dose}}_{\text{NAR}‐\text{NCT}}}\times 100\%. \end{array}$$

## 3. Results and Discussion

### 3.1. NAR-NCT Cocrystal

**Figure 3:**
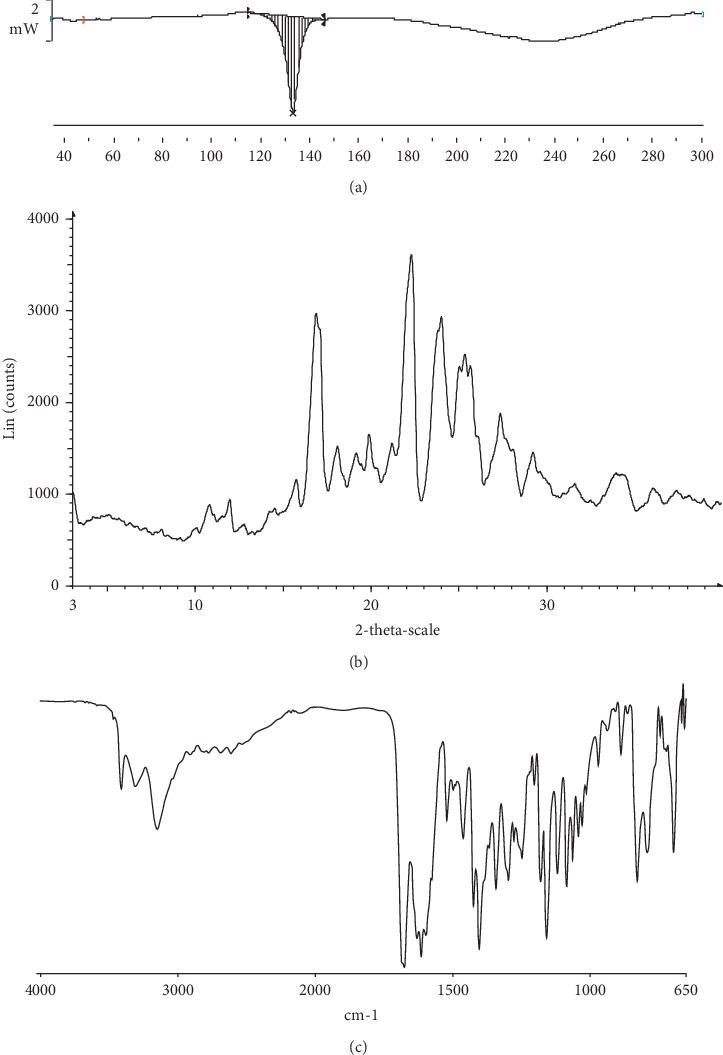
The spectrum of NAR-NCT: (a) DSC, (b) XRPD, and (c) IR.

The NAR-NCT cocrystal was prepared and characterized, which can improve solubility in water. The DSC endothermic peak of the NAR-NCT was 134.68°C. The characteristic peaks in the DSC, XRPD, and IR spectra of NAR-NCT were different from the naringenin-nicotinamide cocrystal reported in the literature [25]. It is shown that a new NAR-NCT cocrystal was formed. The DSC, XRPD, and IR spectra are shown in Figure 3.

### 3.2. Stability of NAR-NCT

**Figure 4:**
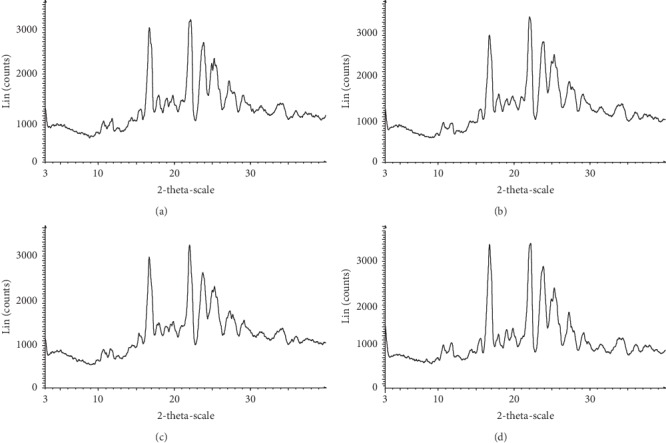
The XRPD pattern of NAR-NCT (a) 60°C for 10 days, (b) relative humidity of 90 ± 5% for 10 days, and (c) light of 5000 Lx for 10 days, (d) 6 months at room temperature.

The stability of NAR-NCT was investigated at 60°C, at a relative humidity of 90 ± 5%, under light of 5000 Lx for 10 days and after 6 months at room temperature. In terms of appearance, NAR-NCT showed no difference under the above conditions. A comparison of the XRPD spectra (Figure 4) showed that the overall peak shape, position, and intensity are not a significant difference, indicating that the NAR-NCT was stable at 60°C, at a relative humidity of 90 ± 5%, under light of 5000 Lx for 10 days and after 6 months at room temperature.

### 3.3. Optimization of the Plasma Sample Preparation

**Figure 5:**
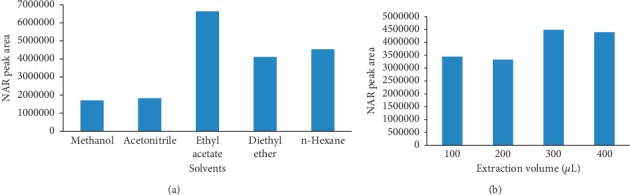
Effects of solvents on NAR extraction: (a) solvent selection and (b) ethyl acetate volume.

Protein precipitation and liquid-liquid extraction methods were tested for the removal of interfering species from the sample. Organic solvents, such as methanol, acetonitrile, diethyl ether, ethyl acetate, and n-hexane were investigated, and the results are shown in Figure 5. The extraction effect of ethyl acetate was the best, so the extraction effects of 100, 200, 300, and 400 *μ*L of ethyl acetate were analysed. Results showed that ethyl acetate (300 *μ*L) had the best extraction effect. Therefore, ethyl acetate (300 *μ*L) was used to the extracted solvent for pretreatment.

### 3.4. Method Validation

**Figure 6:**
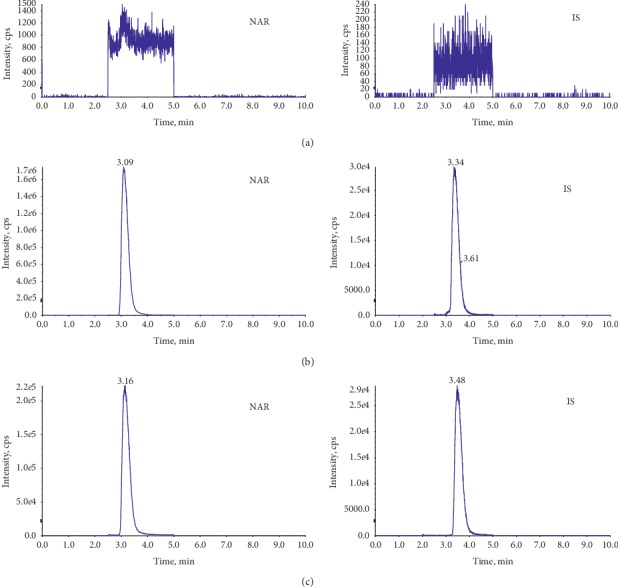
The chromatograms of MRM: (a) blank plasma, (b) plasma mixed with NAR, and (c) plasma after oral administration of NAR.

The blank plasma sample showed no signals interfering with the measurement of the analyte. NAR and IS were well separated from endogenous substances and metabolites, and no interference was observed. The chromatograms are shown in Figure 6.

The calibration curve showed good linearity, at the range of 1.03–821 ng/mL, *Y* = 0.11*X* + 0.0914, and *r* = 0.9995. The standard error of slope and intercept was 0.002 and 0.005, respectively. LLOQ was 1.03 ng/mL for the NAR plasma sample.

The results of precision and accuracy during intraday and interday were as follows: both RSD (%) values were ≤8.5%, while both RE (%) values were at the range of −6.2% to 4.7% and −2.6% to 2.8%, respectively (Table 2).

The extraction recoveries were 69.0% to 76.5%, and RSD (%) values were ≤10.2%. The matrix effects were 107.7% to 131.2%, and RSD (%) values were ≤7.7% (Table 2).

The plasma samples were stable for 24 h at room temperature (25 ± 3°C) with the RE% value of 0.9% to 10.3%. After three freeze-thaw cycles, NAR also remained stable (RE%: 1.6% to 4.8%). The sample exhibits 21 days of long-term stability (RE%: −6.9% to 0.2%) at −80°C, and the ready-to-inject sample was stable for 24 h in an autosampler at 25°C (RE%: −8.4% to 4.4%). These results are recorded in Table 2.

After the plasma QC samples were diluted with blank plasma, the RE ranged from −8.1%% to 1.9%, and the RSD was 4.7% (Table 3). These results met the requirements of methodological validation.

### 3.5. Pharmacokinetics and Bioavailability

**Figure 7:**
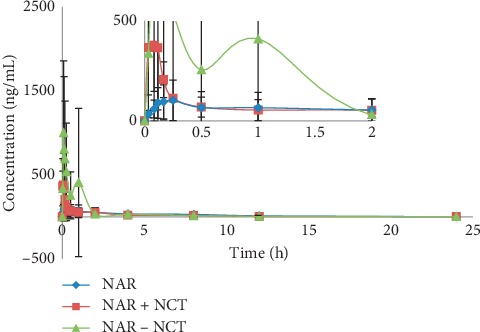
Drug concentration-time curve (in terms of NAR 30 mg/kg) (*n* = 6).

The plasma concentrations of NAR were determined by LC-MS/MS. The concentrations were analysed with DAS Software. The main pharmacokinetic parameters are recorded in Table 4, and the mean plasma concentration versus time curves for NAR is shown in Figure 7.

Compared with NAR, the C_max_ of NAR + NCT was greater by a factor of 2.06, and T_max_ changed from 0.49 h to 0.11 h. Although NAR + NCT accelerated drug absorption, it did not improve drug metabolism. The values of CL and *t*_1/2_ showed that the elimination rates of the two groups were similar. As a result, the relative bioavailability was 77.23% of NAR.

A comparison between NAR-NCT and NAR showed that C_max_ was increased by a factor of 8.43, and T_max_ was advanced from 0.49 h to 0.09 h. In contrast to NAR + NCT, the CL of NAR-NCT decreased from 91.1 L/h/kg to 49.1 L/h/kg, and *t*_1/2_ increased from 5.37 h to 8.24 h, showing the rapid absorption and slow elimination of this formulation. The relative bioavailability of NAR-NCT was 175.09% of NAR, compared with 80.89% reported in the literature [25]. This result showed that NAR-NCT can improve bioavailability and has the advantages of fast absorption and slow elimination. The above phenomenon conformed to the hypothesis [26]. NCT was easily soluble in water, and in aqueous biological environments, NCT was more easily detached from the crystal lattice and readily entered into solution. Then, the NAR particles remained in the solution medium. Because the rate of NAR-NCT dissolution was faster than the formation of supramolecular aggregates of the NAR molecule itself, C_max_ increased and T_max_ decreased. After the dissolution of NCT, the amorphous NAR, with high free energy, slowly converted to a metastable state and then to the lowest energy NAR configuration (usually the pure drug). The time required for this process resulted in slower NAR elimination and increased AUC in the body.

The peak time of NAR, NAR + NCT, and NAR-NCT is 0.49 h, 0.11 h, and 0.09 h, respectively, so NAR has reached the peak at 0.5 h. And the last points of the pharmacokinetic study in the three groups were less than 1/10 of C_max_, respectively. After 0.5 h, a longer interval of blood collection was set. The rats could be allowed to drink water freely in longer blood sampling intervals. Therefore, the process of blood sampling did not have a serious impact on the pharmacokinetics of NAR.

Only the drug-time curve of NAR-NCT showed the double peaks, which was consistent with enterohepatic circulation [27] and extended NAR action time in the body.

## 4. Conclusions

We investigated the differences in pharmacokinetic characteristics and the relative bioavailability of NAR after oral administration of NAR, NAR + NCT, and NAR-NCT in rats. These results show that NAR-NCT was a viable method for improving the bioavailability of NAR. These results provide important references for the application and research of NAR in the future.

## Figures and Tables

**Table 1:** Mass spectrometry parameters.

Parameters	Value	Parameters	Value
MRM ion pair of NAR	*m*/*z* 271.1 ⟶ 151.0	Declustering potential (DP)	−10 V
MRM ion pair of IS	*m*/*z* 301.2 ⟶ 164.1	Collision energy (CE)	−25 V (NAR)-30 V (IS)
Entrance potentials (EP)	−10 V	Collision Exit Potentials (CXP)	−8 V
Temperature	550°C	Ion spray voltage	−4500 V
Curtain gas	28 psi	Nebulizer gas	50 psi
Turbo gas	50 psi	Dwell time	100 ms

**Table 2:** Method validation of NAR in rat plasma (*n* = 6).

			NAR concentration (ng/mL)			
			1.03	3.08	410	616
Precision accuracy	Intraday	RE (%)	1.4	−6.2	4.7	3.4
		RSD (%)	8.3	8.5	3.2	4.3
	Interday	RE (%)	2.8	−2.6	−0.5	−1.4
		RSD (%)	1.7	6.2	6.9	6.3

Recovery		R (%)	69.0	69.5	69.9	76.5
		RSD (%)	9.0	6.5	3.7	10.2

Matrix effect		ME (%)	111.4	131.2	116.9	107.7
		RSD (%)	7.7	5.3	2.6	1.1

Stability	Short-term (24 h)	RE (%)	3.5	10.3	0.9	1.7
		RSD (%)	6.9	3.2	7.8	0.5
	Freeze-thaw cycles	RE (%)	1.8	2.8	4.8	1.6
		RSD (%)	11.1	12.2	3.9	2.3
	Long-term (21 days)	RE (%)	0.2	−6.5	0.1	−6.9
		RSD (%)	6.2	10.1	5.1	2.1
	Autosampler (24 h)	RE (%)	2.0	4.4	−8.4	−8.3
		RSD (%)	5.4	9.3	3.6	4.0

**Table 3:** Dilution reliability of NAR plasma samples (*n* = 6).

Compound	Nominal concentration (ng/mL)	Dilution factor	Measured concentration (ng/mL)	RE (%)	RSD (%)
NAR	6156	10	599	−2.8	4.7
			566	−8.1		
			628	1.9		
			573	−7		
			626	1.6		
			571	−7.3		

**Table 4:** Noncompartmental pharmacokinetic parameters of NAR after oral administration.

	NAR	NAR + NCT	NAR-NCT
*T *_max_ (h)	0.49 ± 0.7	0.11 ± 0.05^*∗∗∗∗*^	0.09 ± 0.02^*∗∗∗∗*^
*C *_max_ (ng/mL)	120.8 ± 95.0	494.0 ± 427.4^*∗∗*^	1018.3 ± 853.4^*∗∗∗*^
*t *_1/2_ (h)	5.37 ± 2.54	5.50 ± 2.44	8.24 ± 6.88^*∗*^
CL/F (L/h/kg)	91.1 ± 58.0	86.4 ± 48.0	49.1 ± 22.5
V/F (L/kg)	575.1 ± 259.6	580.8 ± 165.0	670.5 ± 766.8^*∗*^
MRT_(0-*t*)_ (h)	5.4 ± 0.9	4.2 ± 1.0	3.9 ± 1.9
MRT_(0-∞)_ (h)	6.8 ± 2.5	5.3 ± 2.2	8.0 ± 9.1^*∗*^
AUC_0-*t*_ (ng/mL^*∗*^h)	509.4 ± 485.2	393.4 ± 135.6^*∗*^	891.9 ± 963.0
AUC_0-∞_ (ng/mL^*∗*^h)	567.6 ± 604.0	413.8 ± 156.6^*∗*^	936.4 ± 943.1
Fr%		77.23	175.09

^*∗∗∗∗*^ *P* < 0.0001, ^*∗∗∗*^*P* < 0.001, ^*∗∗*^*P* < 0.01, and ^*∗*^*P* < 0.05.

## Data Availability

All data included in this study are available upon request from the corresponding author.
